# Polyomavirus in Saliva of HIV-infected Children, Brazil 

**DOI:** 10.3201/eid1901.120563

**Published:** 2013-01

**Authors:** Tatiana F. Robaina, Gabriella S. Mendes, Fabrício J. Benati, Giselle A. Pena, Raquel C. Silva, Miguel A.R. Montes, Renata Otero, Gloria F. Castro, Fernando P. Câmara, Norma Santos

**Affiliations:** Author affiliation: Federal University of Rio of Janeiro, Rio de Janeiro, Brazil

**Keywords:** Polyomavirus, JCPyV, BKPyV, WUPyV, KIPyV, viruses

**To the Editor:** Human polyomaviruses (HPyVs) are members of the family *Polyomaviridae*. Nine distinct PyVs can infect humans: BKPyV, JCPyV, WUPyV, KIPyV, MCPyV, TSPyV, HPyV6, HPyV7, and HPyV9 (*1*). Primary infections generally occur early in life, are typically subclinical, and are followed by persistence of the virus in the person. Reactivation of infection has been associated with disease in immunocompromised persons (*2*–*6*). We detected the excretion of HPyV in the saliva of HIV-infected children and compared this finding with its prevalence in healthy control children to evaluate the possible association between viral infection and the stage of immunodeficiency.

Samples were collected during August 2009–June 2011 from patients attending the School of Dentistry of the Federal University of Rio de Janeiro, Brazil. Saliva samples were obtained from 60 HIV-infected children (27 (44.9%) boys, 33 (55.1%) girls), 6–13 years of age (median 9.5 years), and 60 healthy children (47.9% male, 52.1% female), 7–12 years of age (median 9.04 years). The study protocol was approved by the Ethics Committee of the Hospital Universitário Clementino Fraga Filho/University of Rio de Janeiro. The parents of all children involved in the study gave informed consent.

Virus DNA was extracted by using the Wizard Genomic DNA Purification Kit (Promega, Madison, WI, USA). Specimens were tested for HPyV by real-time PCR (*7*,*8*). For BKPyV, we used primer pair BKV-F: 5′-GGTGTAGATCAGAGGGAAAGT-3′ and BKV-R: 5′-TTGCCAGTG ATGAAGAAG C-3′. Statistical significance was assessed by p<0.05.

HPyVs were detected in 17 (28.3%) and 6 (10%) of HIV-infected and control children, respectively (Table). A higher frequency of viral infection was observed in the HIV-infected group (p = 0.01). Frequency of KIPyV infection was significantly higher among immunocompromised children (p = 0.02). No difference was observed for BKPyV, JCPyV, or WUPyV. The virus loads were similar in both groups (data not shown).

**Table:** HPyVs detected in saliva from HIV-infected and healthy control children, Brazil*

Virus	No. (%) HIV-infected children, n = 60	No. (%) control children, n = 60
BKPyV	3 (5.0)	0
JCPyV	2 (3.3)	4 (6.6)
WUPyV	1 (1.7)	1 (1.7)
KIPyV	8 (13.3)	0
BKPyV+KIPyV	1 (1.7)	1 (1.7)
JCPyV+KIPyV	2 (3.3)	0
Total	17 (28.3)	6 (10.0)

*HPyV, human polyomavirus.

.

HIV-infected persons were classified into 3 immunologic categories: no evidence of immune suppression (CD4^+^ >500 cells/µL; n = 38), moderate suppression (CD4^+^ 200–499 cells/µL; n = 13), and severe suppression (CD4^+^ <200 cells/µL; n = 9). HPyV was more frequently detected among children with severe immunosuppression (n = 7; p<0.001). However, no significant correlation was observed between the frequency of HPyV DNA detection and the use of highly active antiretroviral therapy (HAART) (p = 0.156).

Because the immunosuppressed population is increasing around the world, the role of HPyVs as opportunistic pathogens in these persons has become a great concern (*2*–*6*). In this study, we found that the frequency of HPyV infections was higher among HIV-infected children than among the general pediatric population, although infection was not associated with the person’s CD4^+^ cell count. The viral loads were similar in both groups, suggesting that efficiency of viral replication is not related to the person’s immune status. None of the HPyV-positive children, including those with severe immunosuppression (data not shown), showed any symptoms of illness associated with these viruses, such as urinary tract, neurologic, or respiratory tract infection. 

Previous studies analyzed the occurrence of HPyV infections in immunosuppressed persons with AIDS. Sharp et al. investigated the presence of WUPyV, KIPyV, and MCPyV in lymphoid tissue samples from persons with AIDS and healthy controls and found a much higher frequency of infection in the immunosuppressed group (*9*). Babakir-Mina et al. investigated the frequency KIPyV and WUPyV in blood of HIV-1–infected patients compared with blood donors and demonstrated that WUPyV infection was more frequent in HIV-infected patients but the frequency of infection for KIPyV was similar in both groups; they also found no association between CD4^+^ cells count and HPyV infection (*2*). Machado et al. investigated the urinary excretion of BKPyV and JCPyV among HIV-1–infected children and adolescents and healthy controls and demonstrated a significantly higher BKPyV viruria in HIV-infected patients. No difference was observed for JCPyV excretion, however, and no association was found between CD4^+^ values and viral shedding (*3*). Jeffers et al. assessed the salivary shedding of BKPyV on a cohort of healthy and HIV-immunosuppressed persons and found that BKPyV DNA levels in the saliva were significantly higher in HIV-infected patients. They also demonstrated the ability of a BKPyV to replicate in vitro in salivary gland cells and suggested that salivary glands may constitute a reservoir for BKPyV (*10*). Jeffers and Webster-Cyriaque, while investigating the contribution of viral infection to the pathogenesis of salivary gland diseases, detected BKPyV shedding in the saliva of HIV-positive patients with salivary gland diseases more often than in healthy controls and suggested that it played a possible role in the disease (*4*). In contrast, other studies did not detect BKPyV or JCPyV in saliva of either HIV-infected or healthy controls (*6*,*7*).

In this study, we detected DNA of BKPyV, JCPyV, WUPyV, and KIPyV in saliva samples of both HIV-positive and healthy control children, although the frequency of infection was significantly higher among the HIV-infected children. These findings suggest that saliva may be a route of HPyV transmission and that the oral cavity could be a site of virus replication and persistence.
